# Morphology analysis of the C2 pediculoisthmic component and feasibility of safe C2 pedicle screw placement: comparison of multiplanar reconstruction versus traditional radiographic methods

**DOI:** 10.1186/s13018-023-03727-3

**Published:** 2023-03-28

**Authors:** YueLin Wu, ZhaoQuan Liang, JunHao Bao, Ling Wen, Li Zhang

**Affiliations:** 1 The Spine Department, Orthopaedic Center, Guangdong Second Provincial General Hospital, Guang Zhou, Guangdong Province China; 2 The Second School of Clinical Medicine, Southern Medical University, Guangzhou, Guangdong Province China

**Keywords:** High-riding vertebral artery, Multiplanar reconstruction, C2 pediculoisthmic component, C2 pedicle screw, Cervical spine

## Abstract

**Background:**

Preoperatively evaluating the feasibility of safe C2 pedicle screw placement is the key to avoiding iatrogenic vertebral artery injury. However, it has not been verified whether the conventional CT measurements of C2 pediculoisthmic component (PIC) are reliable and accurate, and the results may lack validity. The purpose of this study is to analyze the evaluative performance of conventional CT measurements and to create an accurate predictor of morphometrics of C2 PIC.

**Methods:**

A total of 304 C2 PICs were measured in 152 consecutive patients who underwent CT examination of the cervical spine between April 2020 and December 2020. We obtained the morphometric parameters of C2 PIC by measuring minimum PIC diameter (MPD) in CT multiplanar reconstruction versus conventional measurements of transverse PIC width (TPW), oblique PIC width (OPW) and definition of high-riding vertebral artery (HRVA). The outer diameter measured less than 4 mm in MPD was regarded as the standard of precluding safe C2 pedicle screw insertion. The evaluative performance of the conventional CT measurements was assessed, and the correlation between conventional CT measurements and measurements in CT multiplanar reconstruction was calculated.

**Results:**

The parameters in OPW and MPD were measured significantly larger than those in TPW, and the preclusion of C2 pedicle screw placement evaluated from TPW and HRVA was significantly higher than that evaluated from OPW and MPD. The sensitivity of TPW was 93.09%, and the specificity was 79.31%. The sensitivity and specificity of OPW were 97.82% and 82.76%. The sensitivity of HRVA was 88.36%, and the specificity was 96.55%. Strong agreement with the highest correlation coefficient (0.879) and determination coefficient (0.7720) suggested that the outer diameter of OPW could be useful for the precise prediction of MPD.

**Conclusions:**

CT MPR allows accurate measurement of the narrowest section of the C2 PIC. The outer diameter of OPW could be simply measured and be useful for precise prediction of MPD, which makes C2 pedicle screw placement more safely than the conventional measurement of TPW and HRVA.

## Introduction

Atlantoaxial instability may lead to severe neural and psychological injuries due to cervical spinal cord compression. Posterior atlantoaxial internal fixation can be used to fix and stabilize the occipitocervical junction to prevent neurologic damage [1, 2].

Multiple techniques of posterior surgical fixation at the second cervical vertebra (C2) include transarticular screws, pedicle screws, pars screws, and more recently, translaminar screw fixation reported by Wright et al. [3]. Transarticular screw fixation was once considered the gold standard for posterior atlantoaxial internal fixation, but nearly 20% of patients are at risk of a damaging vertebral artery due to the high-riding vertebral artery (HRVA) [4, 5]. C2 pars screw fixation and C2 translaminar screw fixation are usually used as alternative techniques for the failure of C2 pedicle screw fixation because of biomechanical deficiency [6, 7]. Thus, pedicle screw fixation at C2 becomes the main procedure owing to the biomechanical advantage of three-dimensional fixation and short-segment fixation.

The key to a successful transpedicular procedure is that the small pedicle is safely penetrated. Otherwise, severe complications, such as iatrogenic vertebral artery injury, may occur, which can lead to potentially catastrophic results such as vertebrobasilar insufficiency, brain stem and posterior fossa infarction, and even death. Several studies have investigated the morphometry of the C2 pedicle isthmus. Many CT or CTA methods have been used to measure the morphometric parameters of the C2 pedicle isthmus to evaluate the feasibility of safe C2 pedicle screw or transarticular screw placement, including transverse C2 pedicle width, the definition of HRVA, variable angle interpolation of axial CT scans (oblique CT scans) according to Yin et al. [8] and so forth [9–12]. However, because of the complicated space position, the C2 pedicle width cannot be measured accurately from the transverse plane and pedicle height from a sagittal plane in CT images. The orthogonal planes may be biased for suboptimal acquisition techniques or patient-related factors, such as deformity, rotation or dislocation of vertebral bodies [13]. It has not been verified whether the conventional parameters are reliable and accurate, and the results may lack validity.

Yuan et al. [14] studied the morphology of the C2 pedicle isthmus using multiplanar reconstruction (MPR) CT images and defined the C2 pediculoisthmic component (PIC) concept. The C2 PIC section is the narrowest section and the bottleneck of the C2 pedicle isthmus, which is entirely vertical to the pedicle axis and was defined as the line bisecting the pedicle isthmus on the axial and sagittal planes. Although studies found that the MPR measurement matches the width of the C2 PIC remarkably [15–17], the complex measurement method has low clinical practicability. In addition, little is known about the discrepancy and relationship between the PIC diameter measured from MPR-CT images and the pedicle width measured from axial CT images and oblique CT images.

The purpose of this study was to analyze the feasibility of safe C2 pedicle screw placement by measuring the morphometric diameters with different CT method, and to create an accurate predictor of PIC morphology based on conventional CT measurements.

## Materials and methods

### Subjects

We enrolled consecutive patients who underwent CT examination of the cervical spine at our hospital between April 2020 and December 2020. Patients with rheumatoid arthritis, ankylosing spondylitis, spinal metastasis or congenital cervical spine fusion, such as Klippel–Feil syndrome, and patients who had previously undergone head or cervical spine operation were excluded.

### Data collection and analysis

The DICOM data collected from a 256-slice multidetector computed tomography scanner (iCT-256 Philips Healthcare, Amsterdam) were imported into DICOM viewer software (RadiAnt DICOM Viewer, Medixant, Poznan, Poland). The settings used for the CT scans were as follows: helical mode; slice thickness, 0.4–2 mm; tube voltage, 100 kV; tube current, 340 mA; window center, 800; and window width, 2,000. We used the volume rendering (VR) and multiplanar reconstruction (MPR) functions of RadiAnt software. The linear precision of the measurements using the software was 0.01 mm. Safe C2 pedicle screw placement was not considered feasible in pedicles with < 4 mm outer diameter measured by conventional CT methods and CT multiplanar reconstruction.

#### Conventional measurements were obtained as follows

##### Transverse PIC width (TPW)

**Fig. 1:**
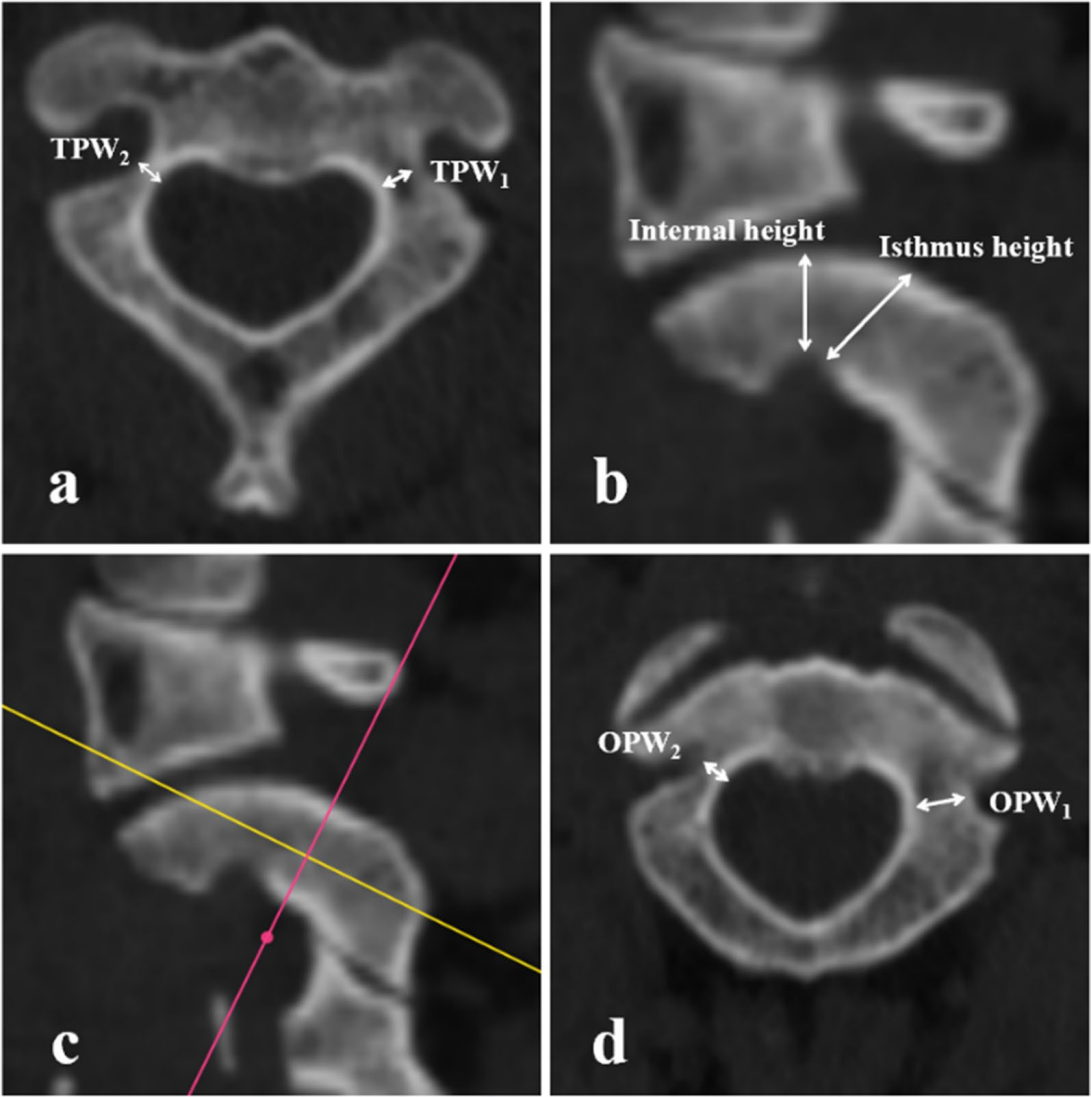
**a** The diameter of the endosteal cavity (*TPW*_*1*_) and outer diameter (*TPW*_*2*_) were measured perpendicular to the pedicle axis on the orthogonal axial plane. **b** Isthmus height and internal height were measured on an orthogonal sagittal image 3 mm lateral to the cortical margin of the spinal canal wall at C2. **c** and **d** The “MPR” operation in the software's toolbar was performed. The pedicle axis was determined by manipulating the sagittal multiplanar reconstructions. The width of the endosteal cavity (*OPW*_*1*_) and outer diameter (*OPW*_*2*_) were measured at the cross-sectional slices parallel to the sagittal pedicle axis

The width of the endosteal cavity (TPW_1_) and outer diameter (TPW_2_) were measured perpendicular to the pedicle axis on the orthogonal axial plane (Fig. 1a).

##### Internal height and isthmus height for defining HRVA

Isthmus height and internal height were measured on an orthogonal sagittal image 3 mm lateral to the cortical margin of the spinal canal wall at C2. According to Bloch et al. [18] and Neo et al. [10], HRVA is defined as the isthmus height being less than 5 mm or the internal height of the lateral mass being less than 2 mm (Fig. 1b). Safe C2 pedicle screw placement was not considered feasible in pedicles with HRVAs.

##### Oblique PIC width (OPW)

The “MPR” operation in the toolbar of software was performed. The pedicle axis was determined by manipulating the sagittal multiplanar reconstructions. The width of the endosteal cavity (OPW_1_) and outer diameter (OPW_2_) were measured in cross-sectional slices parallel to the sagittal pedicle axis (Fig. 1c, d).

#### Measurements in CT multiplanar reconstruction were obtained as follows:

##### Minimum PIC diameter on the narrowest portion (MPD)

**Fig. 2:**
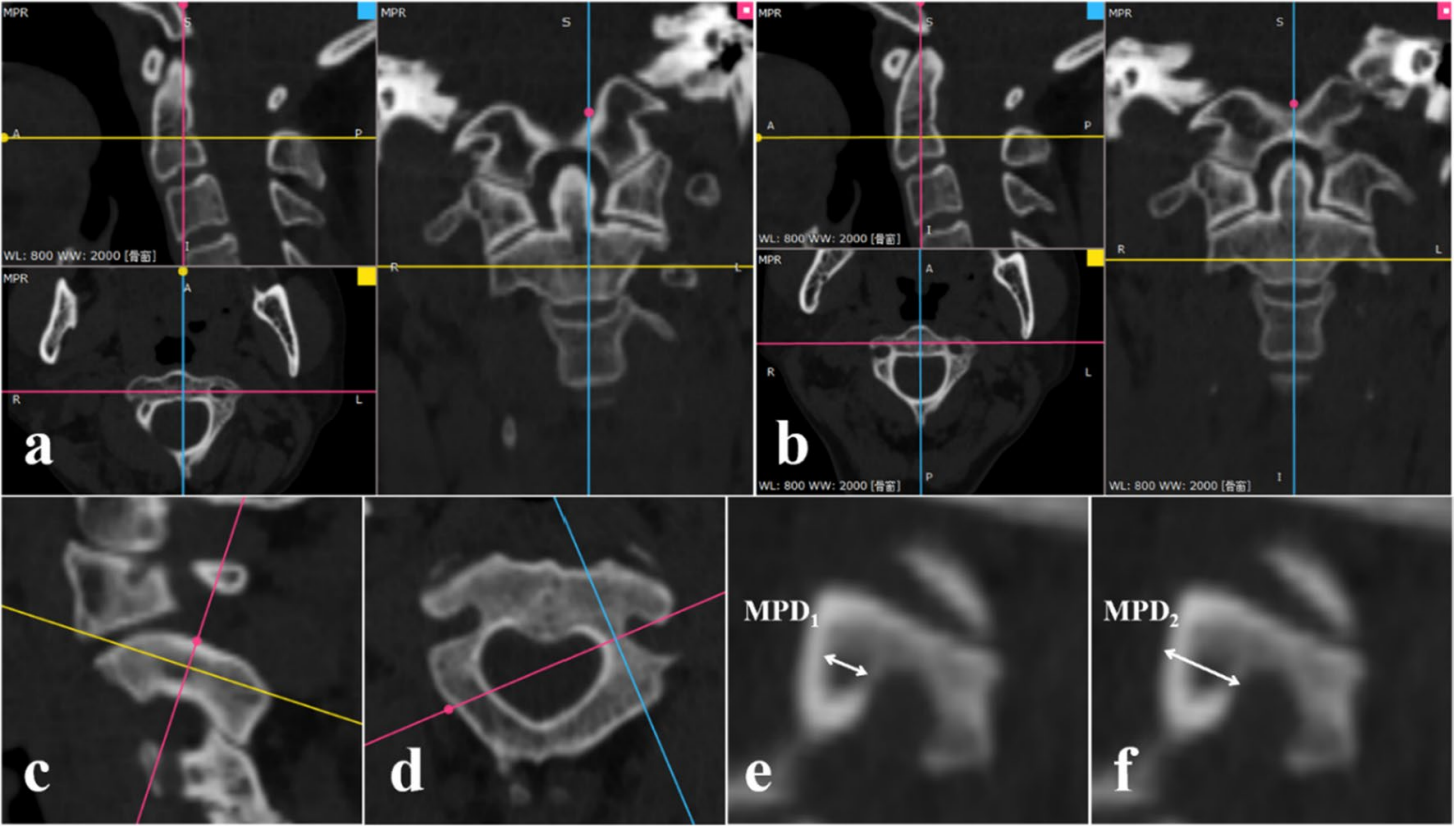
**a** and **b** The “MPR” operation in the toolbar of software was performed. First, possible deformity or inaccurate acquisition techniques were corrected to the actual axial and sagittal plane of C2 vertebrae by manipulating the multiplanar reconstructions. **c** and **d** Then, consecutive cross-sectional slices of the oblique coronal planes perpendicular to the pedicle axis of both the axial and sagittal planes were determined. **e** and **f** Among those consecutive sections, the narrowest portion of the PIC was identified. The widths of the endosteal cavity (*MPD*_*1*_) and outer diameter (*MPD*_*2*_) were measured at the narrowest sections of the PIC

The “MPR” operation in the toolbar of software was performed. First, possible deformity or inaccurate acquisition techniques were corrected to the actual axial and sagittal plane of C2 vertebrae by manipulating the multiplanar reconstructions (Fig. 2a, b). Then, consecutive cross-sectional slices of the oblique coronal planes perpendicular to the pedicle axis of both the axial and sagittal planes were determined (Fig. 2c, d). Among those consecutive sections, the narrowest portion of the PIC was identified. The widths of the endosteal cavity (MPD_1_) and outer diameter (MPD_2_) were measured at the narrowest sections of the PIC (Fig. 2e–f).

### Statistical analysis

Statistical analyses were performed using the IBM SPSS Statistics package (ver. 26.0; IBM Corp.) and GraphPad Prism version 8.0.2 for Windows (GraphPad Software, San Diego, California). A one-way ANOWA test was performed to compare the morphometric parameters of C2 PIC between TPW, OPW and MPD. A paired-samples *t-*test was performed to analyze the difference between the left and right widths of the C2 PIC. Kappa statistics were used to determine the agreement between TPW, OPW, HRVA and MPD, and the McNemar test was used to compare the evaluative accuracy of all methods. Correlational analysis was performed on the relationship between TPW, OPW and MPD. *P* values less than 0.05 were considered to indicate statistical significance.

## Results

### Demographic data

A total of 304 consecutive C2 PICs were measured in 152 patients between April 2020 and December 2020. Eighty-seven were male (57.2%), and 65 were female (42.8%), with an age of 59.36 ± standard deviation (SD) of 13.73 years (range 16–87 years) at the time of CT scanning.

### Radiographic results

**Fig. 3:**
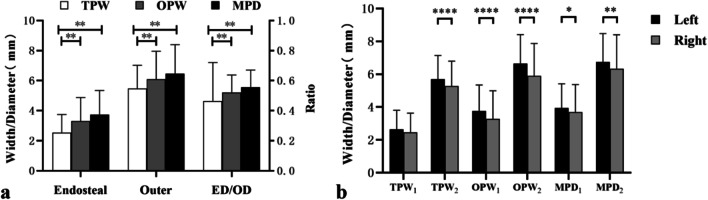
Comparison between the transverse PIC width (*TPW*), oblique PIC width (*OPW*) and minimum PIC diameter (*MPD*) at the parameters of endosteal diameter, outer diameter and the ratio of ED/OD (**a**) and between the left and right widths of the PIC at TPW_1_, TPW_2_, OPW_1_, OPW_2_, MPD_1_ and MPD_2_ (**b**). Error bars show standard deviations, *P < 0.05, **P < 0.01, ****P < 0.0001

All measured data are shown in Table 1. In OPW and MPD, the endosteal diameter, outer diameter and ratio of ED/OD were significantly greater than those in TPW (*P* < 0.01). However, no significant difference was calculated between OPW and MPD (Fig. 3a). A difference in measurement was noted between the right and left PICs (Fig. 3b). The left PIC measurement was significantly larger than the right PIC measurement in OPW and MPD for both endosteal and outer parameters, and in TPW for outer parameters (*P* < 0.05). The HRVA was present in only the right PIC in 22 patients (36.7%), in only the left PIC in 38 patients (63.3%) and in both PICs in 11 patients (18.3%). A significant difference in the frequency of HRVA was noted between the left and right sides (*P* = 0.021).

**Table 1:** Morphometric parameters of C2 PIC measured from transverse PIC width (*TPW*), oblique PIC width (*OPW*) and minimum PIC diameter (*MPD*)

Diameter	Endosteal (mm)	Outer (mm)	ED/OD
*TPW*			
L	2.64 ± 1.16	5.69 ± 1.44*	0.47 ± 0.29
R	2.46 ± 1.16	5.27 ± 1.52	0.46 ± 0.17
Both	2.55 ± 1.16	5.48 ± 1.49	0.46 ± 0.24
*OPW*			
L	3.01 ± 1.22*	5.96 ± 1.65*	0.54 ± 0.11
R	2.51 ± 1.19	5.15 ± 1.60	0.52 ± 0.13
Both	2.76 ± 1.23	5.56 ± 1.67	0.53 ± 0.12
*MPD*			
L	3.94 ± 1.48*	6.74 ± 1.73*	0.57 ± 0.10
R	3.69 ± 1.68	6.34 ± 2.06	0.55 ± 0.12
Both	3.82 ± 1.58	6.54 ± 1.91	0.56 ± 0.12

*Data are for comparison with R (*P*<0.05)

The comparison of the evaluative feasibility of safe C2 pedicle screw placement is shown in Tables 2 and 3. The preclusion of C2 pedicle screw placement evaluated from TPW and HRVA was significantly higher than that evaluated from OPW and MPD (*P* < 0.05).

**Table 2:** Comparison of preclusion of C2 pedicle screw placement between TPW, OPW, MPD and HRVA

	Feasibility of screw placement	
	+	−
TPW	262	42
OPW	274	30
MPD	275	29
HRVA	244	60

+  = feasible for safe C2 pedicle screw placement

−  = risky for C2 pedicle screw placement

**Table 3:** Comparison of evaluative performance between TPW, OPW, HRVA and the criteria for the feasibility of safe C2 PS placement based on MPD

		MPD	
		+	−
TPW	+	256	6
	−	19	23
OPW	+	269	5
	−	6	24
HRVA	+	243	1
	−	32	28

+  = feasible for safe C2 pedicle screw placement

− = risky for C2 pedicle screw placement

Agreement on the feasibility of C2 pedicle screw insertion between TPW and MPD was moderate (91.78% [279 of 304 PICs]; *κ* = 0.60 [95% CI: 0.46, 0.74], *P* < 0.001; McNemar *P* = 0.015). The sensitivity of TPW was 93.09%, and the specificity was 79.31%. Agreement on the feasibility of C2 pedicle screw insertion between OPW and MPD was strong (96.38% [293 of 304 PICs]; *κ* = 0.80 [95% CI: 0.68, 0.91], *P* < 0.001; McNemar *P* > 0.1). The sensitivity of OPW was 97.82%, and the specificity was 82.76%. Agreement on the feasibility of C2 pedicle screw insertion between the HRVA and MPD was weak (89.14% [271 of 304 PICs]; *κ* = − 0.197 [95% CI: − 0.27, − 0.12], *P* < 0.001; McNemar *P* < 0.001). The sensitivity of HRVA was 88.36%, and the specificity was 96.55%.

### Correlation result

**Fig. 4:**
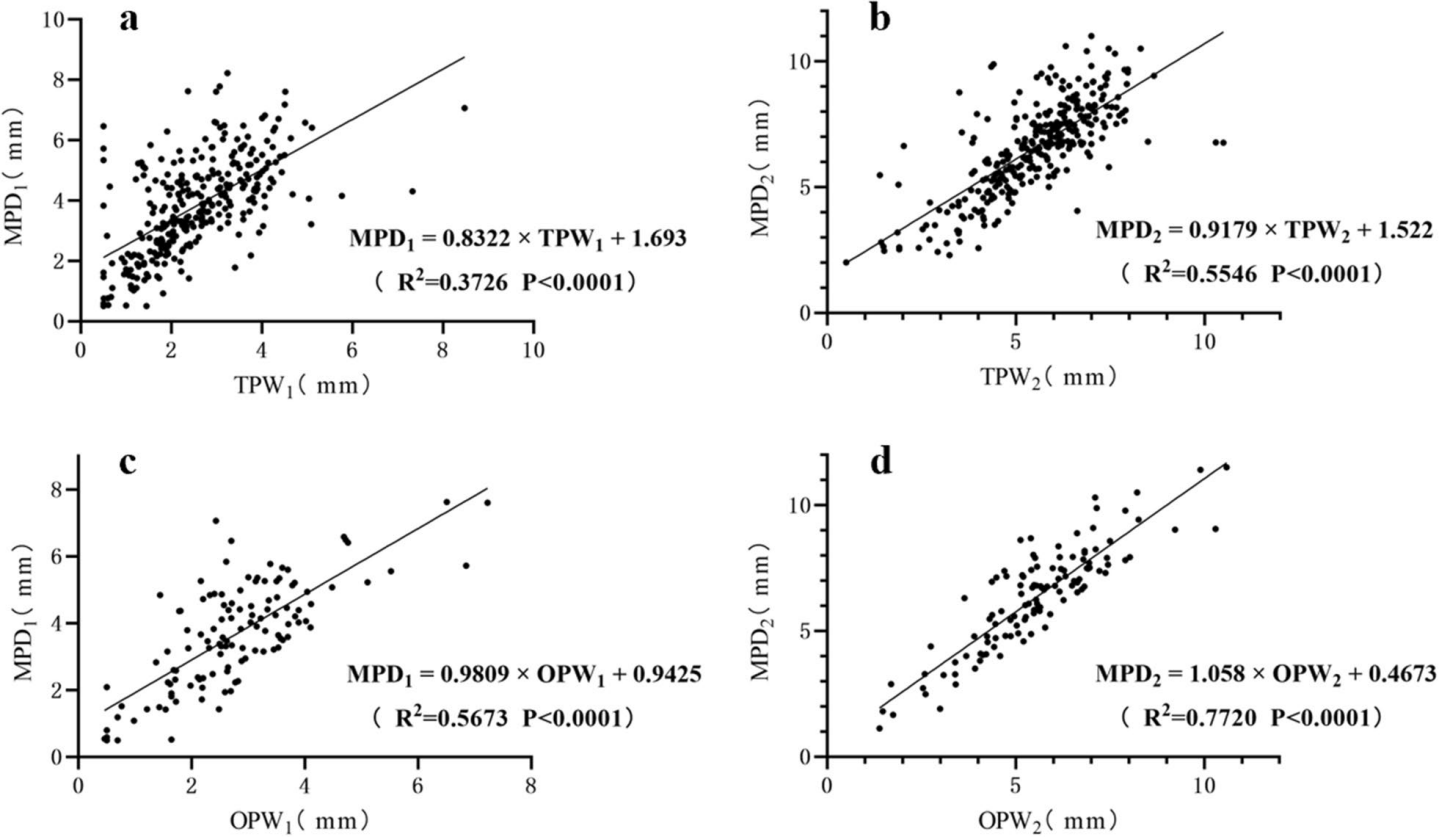
**a** and** b** Correlation between the minimum PIC diameter (*MPD*) and transverse PIC width (*TPW*) at the endosteal diameter and outer diameter, **c** and** d** Correlation between the MPD and oblique PIC width (*OPW*) at the endosteal diameter and outer diameter

A linear regression analysis revealed that parameters measured with MPR-CT had a significant positive linear correlation with the parameters measured by orthogonal axial CT scan and oblique axial CT scan (Fig. 4).

Simple linear regression analysis gave the following relationship between MPD and TPW: MPD_1_ = 0.8322 × TPW_1_ + 1.693 at endosteal diameter (*r* = 0.610, R^2^ = 0.3726, *P* < 0.0001) and MPD_2_ = 0.9179 × TPW_2_ + 1.522 at outer diameter (*r* = 0.745, *R*^2^ = 0.5546, *P* < 0.0001).

Simple linear regression analysis gave the following relationship between MPD and OPW: MPD_1_ = 0.9809 × OPW_1_ + 0.9425 at endosteal diameter (*r* = 0.753, *R*^2^ = 0.5673, *P* < 0.0001), MPD_2_ = 1.058 × OPW_2_ + 0.4673 at outer diameter (*r* = 0.879, R^2^ = 0.7720, *P* < 0.0001).

## Discussion

The presence of an HRVA or narrowed C2 pedicle isthmus poses a great risk when placing a C2 pedicle screw. Preoperative evaluation of the pedicle isthmus size and clarification of the position of the vertebral artery are closely related to anatomic investigation of the C2 pedicle isthmus. Many surgeons recommend using CT images to evaluate the feasibility of C2 pedicle screw placement.

With the development of CT techniques, several studies that focus on the morphology of C2 PICs by means of multiplanar reconstruction CT images have emerged. Marques et al. [16] proposed a “two-step” technique with MPR-CT scans to evaluate the feasibility of C2 pedicle screws preoperatively, and postoperative MPR-CT images were used to analyze the actual screw trajectory. Davidson et al. [17] assessed potential C2 pedicle trajectories by MPR-CT scans with both fixed and variable starting points. In this study, we retrospectively investigated the morphometrics of the C2 PIC with MPR-CT scans, which allows more accurate measurement of the narrowest section of the C2 PIC to evaluate the feasibility of the C2 pedicle for surgical fixation.

In the present study, the mean MPD for endosteal diameter was 3.82 ± 1.58 mm and 6.54 ± 1.91 mm for outer diameter, which were similar to those of Yuan et al. [14], but they were narrower than those of Maki et al. [15], who reported that the average width of the endosteal cavity and the outer diameter of the PIC were 5.6 ± 1.9 mm and 7.2 ± 1.4 mm, respectively. The left PIC measurement was measured significantly greater than the right for both endosteal and outer parameters in our results, but the presence of HRVA was higher in the left PIC than in the right PIC. Ebraheim et al. [19] and Mandel et al. [20] also reported the dominant left width of pedicles. Naderi et al. [21] reported that the right inferior widths of PIC were wider than the left inferior widths, and Yuan et al. [14] reported that differences in parameters between the left and right sides were not significant. The discrepancy between left and right PIC remains controversial. A further investigation should be done to clarify.

Given that 80% of the outer diameter in C2 PIC would be exceeded by the inner diameter of screw rod ranging from 2.4 to 2.9 mm and that screw thread is allowed to cut into part of the PIC cortex [22], we regarded pedicles that were measured less than 4 mm in outer diameter as the index of precluding safe C2 pedicle screw insertion [23]. Our result showed that the ratio of ED/OD in TPW was significantly smaller than those in OPW and MPD, indicating the thinner endosteal diameter and thicker cortical diameter measured in TPW, which means that it is a disadvantage for surgeons to select the appropriate diameter of pedicle screw by measuring TPW.

Following the recommendation of Dull et al. [9] for preoperative oblique axial CT imaging for C1-C2 transarticular screw fixation, Burke et al. [24] reported that the C2 pedicle widths and lengths measured from the axial CT images were significantly smaller than the widths and lengths measured by using oblique axial CT images. Yin et al. [8] further reported that the widths of the C2 pedicles measured from oblique CT scans were 1.4 and 1.5 times higher than their widths measured from axial CT scans. However, little is known about the discrepancy between C2 PIC width measured from MPR-CT images and width measured by orthogonal axial CT images. In our study, the endosteal diameter and outer diameter in TPW were significantly smaller than those in OPW and MPD, and the preclusion of C2 pedicle screw placement evaluated from TPW was significantly higher than that evaluated from OPW and MPD. The result of evaluative performance showed that the sensitivity and specificity of TPW were 93.09% and 79.31%, respectively. This means that 20.69% of C2 PICs that were actually not feasible for safe C2 screw placement may be misjudged by TPW to insert a pedicle screw, while 6.91% of risky C2 pedicle screws evaluated from TPW could actually be placed.

Forty-nine (32.24%) patients had preexisting HRVAs, accounting for 19.74% of all PICs. The sensitivity and specificity of HRVA were 88.36% and 96.55%, respectively. In other words, 3.45% of C2 PICs without HRVA that is actually not feasible for screw placement were misjudged, whereas 11.64% of C2 PICs with HRVA that can actually be placed a screw were omitted.

The risk of vertebral artery injury due to misjudgment of C2 pedicle screw placement may be increased when the feasibility of screw placement is evaluated only from TPW. Conversely, when evaluating only the definition of HRVA, screw placement with relatively insufficient biomechanics, such as C2 pars screws, may be chosen because of preclusion of C2 pedicle screws. This was because the difference in measuring planes resulted from the irregular morphology of C2 PIC. First, the C2 PIC is a unique structure and presents a three‑dimensional inclination with a complicated space position. Xu et al. [25] measured a superior angle of 20.2° ± 3.9° and a median angle of 33° ± 3.2° at the C2 pedicle. Yuan et al. [14] reported that the median angle was 11.1° ± 2.4° (range 7.0°–15.0°) in the superior view of C2 and 42.6° ± 4.9° (range 32°–50.5°) in the inferior view of C2 and explained that the inferior structure of C2, as a transitional vertebra, was similar to that of C3. Second, the typical morphology of the narrowest PIC section was similar to a fishhook, and two kinds of shapes of PICs may be displayed in inferior view: a partially tubular structure or thin paries [14]. Moreover, the orthogonal planes may be biased for suboptimal acquisition techniques or patient-related factors, such as deformity, rotation or dislocation of vertebral bodies. Therefore, the cross section morphology may be changed, resulting in accordingly biased measurement when there is an angle deviation between the MPR-CT plane and the orthogonal plane.

From the perspective of avoidance of vertebral artery injury, it is significant for surgeons to preoperatively estimate an optimal PIC diameter from reconstructed CT images. Our previous research had reported that MPD measurement with CTA multiplanar reconstruction showed the best performance for judging acceptable or unacceptable C2 pedicle screws [26]. However, much work for CT reconstruction and measurement is needed, and there is currently no concise index for optimal C2 PIC diameter calculation based on conventionally measured PIC parameters. In the current study, the highest correlation coefficient (0.879) and determination coefficient (0.7720) suggested that the outer diameter of OPW could be useful for the precise prediction and estimation of MPD. This was because the PIC width measured on an oblique plane parallel to the sagittal pedicle axis could partly offset the deviation of the angle between the measured PIC plane and the actual PIC plane, resulting in error measurement.

The present study is retrospective, has a relatively small sample size and is nonmulticenter. Multicenter study in a larger cohort is needed to validate the results. Despite these limitations, to our knowledge, this is the first report to propose accurate predictors of PIC morphology based on measurements of conventional TPW and OPW for the evaluation of the optimal PIC diameter.

## Conclusions

CT MPR allows accurate measurement of the narrowest section of the C2 PIC. The outer diameter of OPW could be simply measured and be useful for precise prediction of MPD, which makes C2 pedicle screw placement more safely than the conventional measurement of TPW and HRVA.

## Data Availability

The data and materials contributing to this article may be made available upon request by sending an e-mail to the corresponding author.

## Abbreviations

PIC: Pediculoisthmic component
MPD: Minimum PIC diameter
TPW: Transverse PIC width
OPW: Oblique PIC width
HRVA: High-riding vertebral artery
MPR: Multiplanar reconstruction
VR: Volume rendering
